# Talocalcaneal Coalition Resection in the Adult Population: A Systematic Review

**DOI:** 10.7759/cureus.30581

**Published:** 2022-10-22

**Authors:** Alexander A Dermanis, Mohammed Elmajee, Hamza Duffaydar, Ahmad Aljawadi, Shakir Hussain, Anand Pillai

**Affiliations:** 1 Surgery, University Hospitals Birmingham, Birmingham, GBR; 2 Foot and Ankle, Royal Orthopaedic Hospital, Birmingham, GBR; 3 Orthopaedics, University Hospitals Birmingham, Birmingham, GBR; 4 Orthopaedics, Wythenshawe Hospital, Birmingham, GBR; 5 Foot and Ankle, Manchester University National Health Service (NHS) Foundation Trust, Manchester, GBR

**Keywords:** outcomes, resection, coalition, talocalcaneal, adult

## Abstract

Tarsal coalition is a congenital malformation of the tarsal bones of the foot that typically presents with features such as pain, recurrent sprains, and flat foot in childhood. In a small number of patients, a delayed presentation may be apparent, with symptoms instead presenting in adulthood. The most commonly accepted hypothesis is that the tarsal coalition becomes more symptomatic as the coalition progressively ossifies. To this date, no author has systematically evaluated the literature to identify the best approach when surgically managing these patients, in particular concerning the resection of the coalition.

This study aims to systematically review the literature, searching EMBASE, MEDLINE, Web of Science, Google Scholar, and the Cochrane Library to identify and evaluate studies that presented an outcome for resection of the adult talocalcaneal coalition. Alongside overall outcomes, details on the extent of the coalition, surgical method, post-operative regimen, and presenting symptoms were extracted for each patient studied. This was conducted in line with Preferred Reporting Items for Systematic Reviews and Meta-Analyses (PRISMA) guidelines.

With 72 patients, this is the largest evaluation of an adult tarsal coalition population to date. Our findings indicate that talocalcaneal coalition in adulthood presents differently from the classical peroneal spasm found in childhood. Better scores were reported for coalitions either managed with an endoscopic approach or with interposition of the flexor hallucis longus tendon. Despite some reported benefits in the literature, a trial of conservative management or the use of a rehabilitation regimen had a limited impact on the overall patient outcome.

Tarsal coalition in adulthood requires rigorous clinical evaluation to identify appropriate management options. Resection of the coalition is a safe approach to definitively managing these patients, but consideration should be given to the surgical method to ensure each patient has the best outcomes. In particular, consideration should be given to using an endoscopic approach or interposition of the flexor hallucis longus tendon in order to achieve the best patient outcomes. However, there remains a paucity of literature evaluating this demographic and further high-impact studies are required to comprehensively evaluate this population.

## Introduction and background

The talocalcaneal coalition is a musculoskeletal condition characterised by an aberrant connection between the talus and calcaneus bones in the foot, which forms part of a larger group of conditions commonly known as tarsal coalitions. Nearly all talocalcaneal coalitions occur in childhood, most commonly around the ages of 12-15 years [1]. Resection of the talocalcaneal coalition is usually performed in patients who do not have extensive joint degeneration and thus would not be appropriate for a subtalar arthrodesis [2]. Due to a paucity of literature, tarsal coalitions in the adult population are poorly recognised and studied [2], with only a few studies conducted in this population to date. Furthermore, it is also hypothesised by multiple authors that these patients may present with different pathophysiology due to differing rates of coalition ossification [3-5]. This may subsequently have an impact on the management and subsequent outcomes of these patients. This systematic review is designed to evaluate the reported outcomes in the literature specifically for the resection of the talocalcaneal coalition in adults and suggest the best management options available.

## Review

Methods

Using the Preferred Reporting Items for Systematic Reviews and Meta-Analyses (PRISMA) guidelines, we conducted an electronic search using EMBASE, MEDLINE, Web of Science, and the Cochrane Library. In our electronic search, we used the terms: “Treatment”, “Efficacy” “Outcome”, “Results” and “Complications” and combined these with “Tarsal”, “Talocalcaneal”, “Talo-calcaneal” “Talo calcaneal”, “Talarcalcaneal”, “Talar-calcaneal” and “Subtalar”. We then evaluated each paper individually for inclusion in the review. Finally, the reference list of any included papers was searched to try and yield further relevant articles.

Only studies including skeletally mature patients with a symptomatic tarsal coalition, which had resection of the coalition and had reported outcomes relevant to these treatment modalities, were included in this systematic review. To increase the availability of data, case series were also included in our review, however, studies with less than five patients were excluded. Due to differences in operative technique and post-operative care, studies done prior to 1950 were excluded. Any cases involving additional operative interventions not related to tarsal coalition and any inventions with subtalar arthrodesis were excluded. Furthermore, only studies with an English translation were included. Finally, any study that included mixed paediatric, adolescent, and adult groups of patients with inseparable outcomes was excluded.

The quality assessment of these included studies was done according to the Oxford Centre of Evidence-Based Medicine (OCEBM) and The National Institutes of Health (NIH) “Quality Assessment Tool for Case Series Studies” criteria, in order to reduce the risk of bias and assign an appropriate level of evidence for these studies.

We recorded data on patient demographics, past medical history, the extent and pathophysiology of the coalition, histological features, the method of resection, any intervention that was carried out, any associated complications with said intervention, and the overall outcome of the treatment. Outcomes were measured in view of symptomatic relief and functional improvement. The scoring systems studied included the American Orthopaedic Foot and Ankle Score (AOFAS) and visual analogue scales (VAS). Weighted averages were then calculated accordingly and analysed.

Results

**Figure 1:**
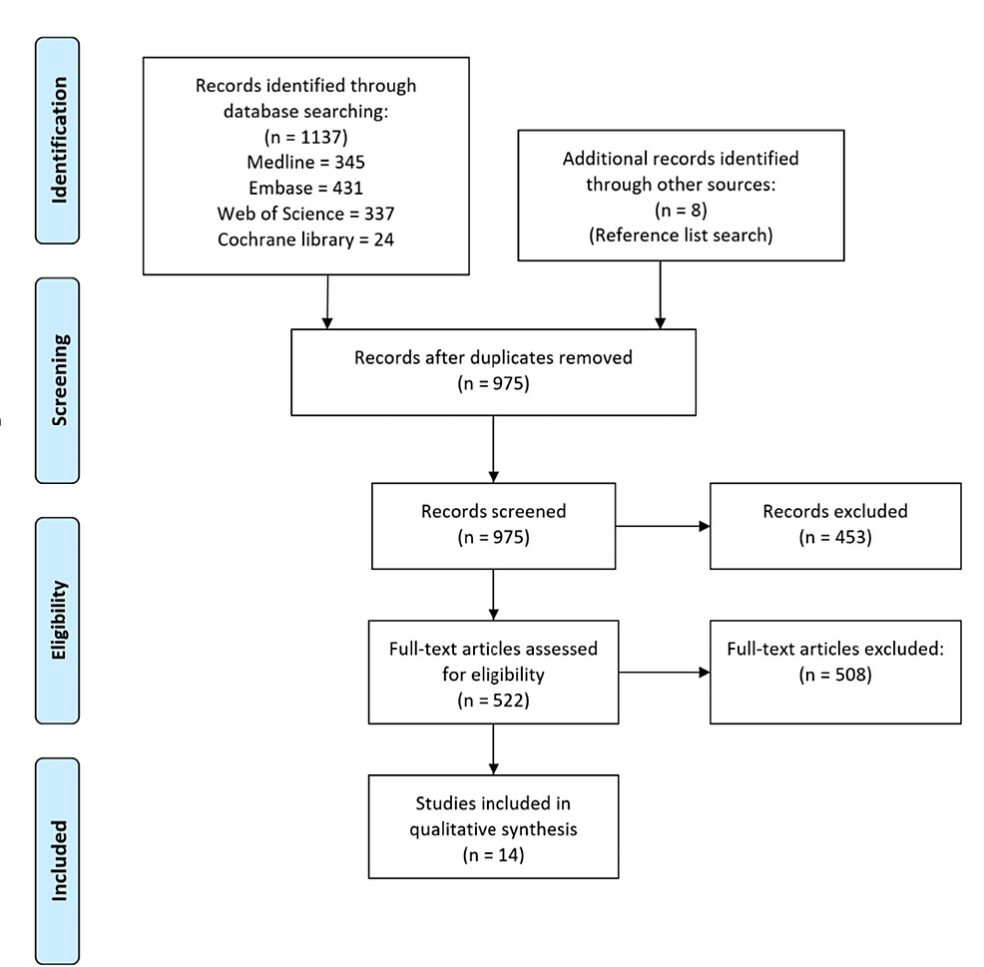
Flow diagram indicating results of the systematic search

Our search initially identified 1137 studies, which were narrowed to 14 studies for inclusion in our review following the removal of duplicates, abstract screening, evaluating the full text of selected studies, and adding any new studies found from the reference lists of the screened studies (Figure 1). Due to recognised heterogeneity, a meta-analysis was not performed.

Baseline characteristics

From the 14 studies included in this review, 72 patients were diagnosed with a talocalcaneal coalition in adulthood, the largest ever adult cohort of patients evaluated with a talocalcaneal coalition. The average age for these patients was 25.8 years (range 16-74 years). Where reported, the majority of patients were male (69.3%), with an approximately even split of left and right feet affected. On average, follow-up occurred over 36.7 months, ranging between 7 to 118 months (Table 1).

**Table 1:** Baseline characteristics

Characteristic	Value	Range
Total number of patients	72 patients, 86 feet	
Average age (years)	25.8 years	16-74 years
Gender (%)	69.4% male, 30.6% female	
Side affected (%)	52.2% right, 47.8% left	
Follow-up (months)	36.7 months	7-118 months

The commonest reported symptom in this cohort was a pain of unspecified origin (31.3%), which was less than expected given this is the commonest presentation in childhood. Interestingly there were very few patients with the classical presentation of peroneal spasm that has been detailed in the literature [6], only 3.13% of patients presented with the features characteristic of peroneal spasm (Table 2). Furthermore, a relatively large proportion of the cohort presented with features of tarsal tunnel syndrome secondary to a coalition (18.8%).

**Table 2:** Presenting symptoms

Primary presenting symptom/constellation of symptoms	Percentage (%)
Pain of unspecified origin	31.3%
Pain in medial malleolar region	12.5%
Sprains	25.0%
Rigidity	9.38%
Tarsal tunnel syndrome (paraesthesia, Tinel’s sign)	18.8%
Peroneal spasm	3.13%

**Figure 2:**
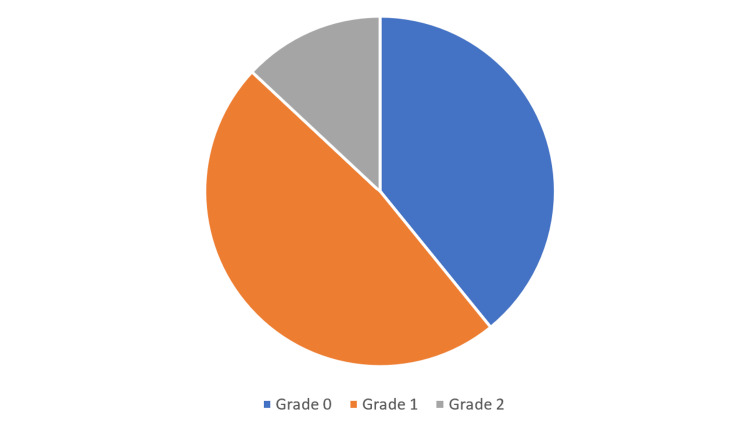
Grading of reported degenerative changes

**Figure 3:**
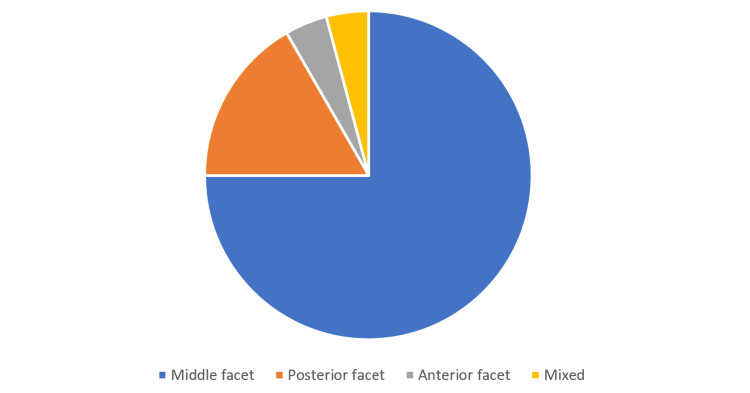
Location of the coalition

All of the patients were found to have an unremarkable past medical history. There was less than 50% involvement of the affected joint in nearly 93% of patients. Where the results of scans were reported, the vast majority of patients had minimal if any degenerative changes of the subtalar joint using the Kellgren and Lawrence classification system (Figure 2). These findings likely demonstrate why resection was preferred over arthrodesis in this cohort. Whilst the data was limited, the commonest location of the coalition was at the middle facet of the subtalar joint (N = 42), followed by the posterior facet (N = 13) and then the anterior facet (N = 2) (Figure 3).

**Figure 4:**
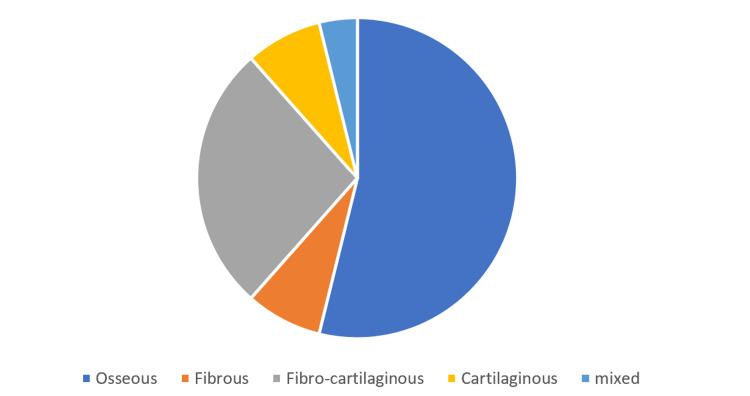
Pathology of the coalition

Where histology was reported, the pathology of the coalition was largely found to be osseous (N = 14), although smaller numbers of fibrous (N = 2) and cartilaginous (N = 2) coalitions were noted (Figure 4). This is largely representative of the literature, which primarily lists osseous coalitions as the commonest pathology across all demographics [7]. 

Overall findings

Given their recognition in the literature and widespread usage in clinical practice, this study primarily identified AOFAS and VAS scores for each study and made relevant comparisons. For all patients who underwent a resection of the talocalcaneal coalition, the average postoperative ankle and hindfoot score (AOFAS score) was 67.7, representing a weighted average increase of 22.7 points (P < 0.05) from the pre-operative average of 45.0. Visual analogue scale (VAS) pain scores reduced from an average of 6.86 to 2.21 (P < 0.05), signifying a change of 4.65. All of the patients studied reported an improvement in their symptoms to some degree postoperatively. Only one complication was reported, namely a case of recurrence of the coalition occurring two years after surgery and hardware removal in a patient who underwent additional procedures alongside resection of the coalition.

Prior conservative management and additional operative interventions

Contrary to the literature that suggests the de facto approach to resection usually follows a trial of conservative management [8], the majority of patients in our cohort did not have a trial of conservative management prior to the resection of the coalition. In general, there was minimal benefit for patients that underwent a trial of conservative management when compared with patients who did not. Table 3 below summarises the outcomes for each group of patients with the talocalcaneal coalition.

**Table 3:** Prior conservative management vs no conservative management

Additional procedures?	Prior conservative management?	Number of feet and name of procedure	AOFAS (pre/postoperative)	Average change in AOFAS score (P < 0.05)
No	No	44 feet	36.2/59.5	23.3
No	Yes	35 feet	64.6/88.4	23.8

Pathology of the coalition

Interestingly the data on the pathology of the coalition was conflicting, with AOFAS scores suggesting that a mixed coalition responded best to surgery, whereas VAS scores indicate that osseous coalitions respond best to surgery. Unfortunately, there was little data available for outcome scores in relation to fibrocartilaginous coalitions (Table 4).

**Table 4:** Outcomes according to the type of coalition resected

Type of coalition	AOFAS (pre/postoperative)	Average change in AOFAS score (P	VAS score (pre/postoperative)	Average change in VAS score (P < 0.05)
Osseous	43.1/66.8	23.7	8.7/1.3	7.4
Fibrous	60.4/97.5	37.1	8.0/2.0	6.0
Fibro-cartilaginous	-	-	-	-
Mixed (osseous, fibrous, cartilaginous)	35.3/75	39.7	6.6/2.3	4.3

Surgical management

Location of Incision and Approach

The importance of careful perioperative planning is paramount. Different approaches to the coalition may influence outcomes due to multiple factors, such as the ability to reach and fully resect the coalition without damaging surrounding structures, the potential for worse scarring, and operator confidence and ability to perform with such an approach [9]. Furthermore, the location of the incision is likely to be influenced by the location of the coalition. In our review, we identified several different approaches to which the talocalcaneal coalition can be visualised. The most popular approach, the medial approach, is typically used in association with middle-facet coalitions. In our review, whilst data was limited, it did seem to suggest that the medial approach did not improve AOFAS scores as high as a posterior approach (Table 5).

**Table 5:** Outcomes according to the location of the incision

Location of the incision	AOFAS (pre/postoperative)	Average change in AOFAS score (P < 0.05)	VAS score (pre/postoperative)	Average change in VAS score (P < 0.05)
Medial	61.9/84.6	22.6	7.5/4	3.5
Posterior	62/94	32	-/6.3	-
Lateral	-/72	-	-/5	-

Interposition of tissue

Commonly coalitions are interposed with tissue following resection. This interposition is hypothesised to prevent recurrence [10] and can vary in tissue from fat, to tendons in the tarsal tunnel themselves. In our study, we identified flexor hallucis longus, extensor digitorum brevis, fat grafting, and fascia lata grafting, albeit in small numbers. The summary of the outcomes for these is presented in Table 6 below:

**Table 6:** Outcomes related to the type of tissue interposed

Interposed tissue	AOFAS (pre/postoperative)	Average change in AOFAS score (P < 0.05)	VAS score (pre/postoperative)	Average change in VAS score (P < 0.05)
Fat Graft	72.5/94.5	22.0	5.5/9.5	4.0
Fascia lata	68.0/90.3	22.3	-	-
Flexor Hallucis Longus	45.5/84.5	39	8.5/1.5	7.0
Extensor Digitorum Longus	-/72	-	-/5	-
No graft	41.5/64.3	22.8	6.43/1.71	4.72

Endoscopic intervention

Consideration must be given to the potential benefits of endoscopic intervention. Studies focusing on endoscopic surgical arthrodesis have demonstrated better outcomes, particularly with respect to functional scores and rates of complications [11]. Our results indicate that for the 20 feet which were identified as having undergone an endoscopic intervention, there was comparatively speaking greater symptomatic improvement with respect to higher AOFAS scores (Table 7).

**Table 7:** Endoscopic vs open approaches

Endoscopic/open?	AOFAS (pre/postoperative)	Average change in AOFAS score (P < 0.05)	VAS score (pre/postoperative)	Average change in VAS score (P < 0.05)
Endoscopic	62.0/94.0	32.0	-/6.3	-
Open	46.0/69.2	23.2	6.86/1.9	4.96

Post-operative regimen

The post-operative regimen generally consisted of an extended programme of exercise and physiotherapy following immobilisation. However, the vast majority (80.6%) of patients did not undertake a formal post-operative regimen and instead initiated weight bearing as tolerated (Table 8).

**Table 8:** Formal rehabilitation regimen vs no formal rehabilitation regimen

	AOFAS (pre/postoperative)	Average change in AOFAS score (P < 0.05)	VAS score (pre/postoperative)	Average change in VAS score (P < 0.05)
Rehabilitation regimen	75.7/90.0	14.3	-	-
No rehabilitation regimen	41.4/66.1	24.7	6.86/2.21	4.65

Discussion

Tarsal coalition in the adult is a rare condition that is poorly studied in the literature. Our findings suggest that overall, the resection of this coalition is a safe procedure and with the right technique can result in notable functional improvement. However, the overall average post-operative AOFAS score of 67.7 may be considered by some clinicians to be an unacceptable score for this intervention. Therefore, our review has identified several alterations that may improve surgical outcomes even further namely- considerations given to the approach, type of tissue interposed, and the use of endoscopy which may influence the management of these coalitions in the future.

It did not seem the case that a trial of conservative treatment in surgically managed coalitions led to a substantial improvement in AOFAS scores when compared with surgical resection alone. This is interesting as it suggests that the mainstay of management, a trial of conservative treatment before resection, may not influence the overall outcome for the patient [8]. However, the benefits of conservative intervention may not be immediately clear from this study. Many patients would have been successfully managed on a conservative regimen beforehand and may never require surgical intervention - this subset of patients was not identified in our study.

Our data on the response for different histological profiles of the coalition to operative management was conflicting and a paucity of data may best explain this disparity, as the vast majority of coalitions in this study were osseous. Some authors hypothesise that coalitions ossify as the patients age [3-5] and this may well explain our findings here given that a skeletally mature population was studied.

Our study found an overall benefit to the posterior approach as opposed to the medial approach. Perhaps the underlying explanation for this is that the commonest approach, the medial approach, faces a conundrum once the skin and overlying fascia is dissected. The tarsal tunnel passes directly over the area of the incision and is a direct obstacle to visualising the coalition. The most common approach is to pass through the overlying structures by retracting the flexor hallucis longus posteriorly and the flexor digitorum longus dorsally. This is the approach that was undertaken in nearly all of the coalitions we observed in this study. However, in nine cases, the coalition had hypertrophied and entrapped the flexor hallucis longus tendon, causing impingement when the ankle was dorsiflexed or plantarflexed. The difficulty in visualising the coalition using this approach may lead to incomplete resection of the coalition and subsequently worse outcomes [12].

In the literature, it is also hypothesised that the posterior approach may be advantageous over a medial approach because of increased perfusion pressure, which allows the creation of sufficient working space for the operation of the instrument only at the coalition site [12]. However, there are shortcomings to this approach too. The flexor hallucis longus and the neurovascular bundle can obstruct an otherwise perfect view of the coalition and this is reflected in some studies such as the study by Keeling and Guyton in which they performed endoscopic FHL decompression in eight cadaveric cases, resulting in three cases of tendon injury. Therefore, caution must be given to avoid potential damage to the underlying structures [12]. It should be recognised that both approaches overall yielded clinically acceptable AOFAS scores for this intervention.

Tissue interposition is recognised in the literature to demonstrate good post-operative outcomes for the paediatric population [13-15]. Our study demonstrates encouraging results with the interposition of grafts for talocalcaneal coalition resection, however, these were the most marked with the interposition of the flexor hallucis longus tendon. There is limited data comparing different grafts alongside no clear conclusion as to why different grafts may outperform each other with respect to overall outcomes. It therefore makes it difficult to draw any conclusions with certainty from the literature and our own study, but our findings should serve as a point of reference for future studies.

The endoscopic approach has gained traction over the past few years due to its less invasive nature and potential for improved outcomes with reduced complications. Our study has shown a higher change in AOFAS scores when compared with open procedures, which supports the possibility of more beneficial outcomes with endoscopic intervention. It should be noted that other novel techniques, such as CT-guided resection of the coalition are similarly gaining traction in this area and may also demonstrate beneficial outcomes in the future [15].

The mixed results for a formal post-operative regimen may have multiple underlying reasons. Firstly, the vast majority of post-operative regimens were poorly defined in the literature, making it difficult to stratify the extent of rehabilitation for a given patient. Secondly, multiple studies did not indicate the degree of compliance with the post-operative regimen. Finally, there was little data on the subjective outcome for these patients, hindering a definite conclusion as to whether these patients experienced symptomatic belief. The authors, therefore, recommend interpreting this dataset with caution as further, well-developed studies are needed to definitively establish a conclusion on the most optimal post-operative regimen.

The benefits of this study are that this is the biggest study of an adult tarsal coalition population to date, it has used reliable and systematic methods to evaluate this population and has thoroughly evaluated this population to make concise and conclusive recommendations for future management of adult talocalcaneal coalition. Different findings related to the surgical method, pathology, approach to resection, and post-operative interventions have been identified, which may influence surgical management in the future.

This study has some limitations. Firstly, there is a paucity in the literature with respect to the surgical method for many of the patients studied, particularly with respect to the approach and interposition of tissues, making definitive conclusions challenging. Secondly, there was little information on patients' lifestyles and how the outcome of coalition resection has changed their activities of daily living. Some outcomes may be less acceptable for an athlete, for example. Unfortunately, symptom scores such as AOFAS and VAS scores cannot account for this. Finally, some lack of detail on the degree of joint degeneration secondary to the coalition also may have implicated results as a confounder, as this can affect the severity of presenting symptoms and the degree of symptom resolution.

## Conclusions

In conclusion, this study has demonstrated that multiple factors may influence the outcome of adult patients presenting with the talocalcaneal coalition. Consideration should be given to the approach to coalition resection, the interposition of tissue, the opportunity for endoscopic resection, and the post-operative regimen following resection. Further studies are necessary to establish the optimum management, but the available data suggests an endoscopic intervention from the posterior approach or interposition of the flexor hallucis longus tendon, in particular, may yield beneficial results. A trial of conservative treatment in these patients is unlikely to make any difference in outcomes for patients who did require an operation. Attention should be given to the location, pathology, and extent of the coalition in order to optimise pre-operative planning and achieve the best available outcomes for these patients.

## References

[1] Guduri V, Dreyer MA (2022). Talocalcaneal coalition. https://www.ncbi.nlm.nih.gov/books/NBK549853/.

[2] Thorpe SW, Wukich DK (2012). Tarsal coalitions in the adult population: does treatment differ from the adolescent?. Foot Ankle Clin.

[3] Varner KE, Michelson JD (2000). Tarsal coalition in adults. Foot Ankle Int.

[4] Carson CW, Ginsburg WW, Cohen MD, McLeod RA, Kitaoka HB (1991). Tarsal coalition: an unusual cause of foot pain—clinical spectrum and treatment in 129 patients. Semin Arthritis Rheum.

[5] HA RI, BE T (1948). Etiology of peroneal spastic flat foot. J Bone Joint Surg Br.

[6] Newman JS, Newberg AH (2000). Congenital tarsal coalition: multimodality evaluation with emphasis on CT and MR imaging. Radiographics.

[7] Lemley F, Berlet G, Hill K, Philbin T, Isaac B, Lee T (2006). Current concepts review: tarsal coalition. Foot Ankle Int.

[8] Hayashi K, Kumai T, Tanaka Y (2014). Endoscopic resection of a talocalcaneal coalition using a posteromedial approach. Arthrosc Tech.

[9] Kothari A, Masquijo J (2020). Surgical treatment of tarsal coalitions in children and adolescents. EFORT Open Rev.

[10] Banerjee S, Gupta A, Elhence A, Choudhary R (2021). Arthroscopic subtalar arthrodesis as a treatment strategy for subtalar arthritis: a systematic review. J Foot Ankle Surg.

[11] King A, Parsons S (2020). Endoscopic resection of tarsal coalitions. Foot Ankle Clin.

[12] Keeling JJ, Guyton GP (2007). Endoscopic flexor hallucis longus decompression: a cadaver study. Foot Ankle Int.

[13] Sperl M, Saraph V, Zwick EB, Kraus T, Spendel S, Linhart WE (2010). Preliminary report: resection and interposition of a deepithelialized skin flap graft in tarsal coalition in children. J Pediatr Orthop B.

[14] Di Liddo PE, Rivard DS, Mehler AS, Wertheimer SJ (2000). Resection of talocalcaneal middle facet coalition. Interposition with a tensor fascia lata allograft: a case report. J Foot Ankle Surg.

[15] Stokman JJ, Mitchell J, Noonan K (2018). Subtalar coalition resection utilizing live navigation: a technique tip. J Child Orthop.

